# A Murine Model of Non-Wear-Particle-Induced Aseptic Loosening

**DOI:** 10.3390/biomimetics9110673

**Published:** 2024-11-04

**Authors:** Vincentius Suhardi, Anastasia Oktarina, Yingzhen Niu, Branden Sosa, Julia Retzky, Matthew Greenblatt, Lionel Ivashkiv, Mathias Bostrom, Xu Yang

**Affiliations:** 1 Department of Orthopedic Surgery, Hospital for Special Surgery, New York, NY 10021, USA; suhardivi@hss.edu (V.S.); bostromm@hss.edu (M.B.); 2 Research Institute, Hospital for Special Surgery, New York, NY 10021, USAoktarinaa@hss.edu (A.O.); ivashkivl@hss.edu (L.I.); 3 Department of Joint Surgery, The Third Hospital of Hebei Medical University, Shijiazhuang 050052, China; 4 Department of Pathology and Laboratory Medicine, Weill Cornell Medicine, New York, NY 10065, USA; 5 Department of Orthopedic Surgery, Weill Cornell Medicine, New York, NY 10021, USA

**Keywords:** osseointegration failure, fibrosis, fibrosis progenitor cells

## Abstract

Background: The current murine models of peri-implant osseointegration failure are associated with wear particles. However, the current clinical osseointegration failure is not associated with wear particles. Here, we develop a murine model of osseointegration failure not associated with wear particles and validate it by comparing the cellular composition of interfacial tissues with human samples collected during total joint arthroplasty revision for aseptic loosening. Materials and Methods: Thirty-two 16-week-old female C57BL/6 mice underwent implantation with a press-fitted roughened titanium implant (Control, *n* = 11) to induce normal osseointegration and a press-fitted smooth polymethylmethacrylate implant (PMMA, *n* = 11), a loosely fitted smooth titanium implant (Smooth-Ti, *n* = 5) or a loosely fitted roughened titanium implant (Overdrill, *n* = 5) to induce osseointegration failure. Pullout testing was used to determine the strength of the bone–implant interface (*n* = 6 of each for Control and PMMA groups) at 2 weeks after implantation. Histology (*n* = 2/group) and immunofluorescence (*n* = 3/group) were used to determine the cellular composition of bone–implant interfacial tissue, and this was compared with two human samples. Results: Osseointegration failure was confirmed with grossly loosening implants and the presence of fibrous tissue identified via histology. The maximum pullout load in the PMMA group was 87% lower than in the Control group (2.8 ± 0.6 N vs. 21 ± 1.5 N, *p* < 0.001). With immunofluorescence, abundant fibroblasts (PDGFRα+ TCF4+ and PDGFRα+ Pu1+) were observed in osseointegration failure groups and the human samples, but not in controls. Interestingly, CD146+PDGFRα+ and LepR+PDGFRα+ mesenchymal progenitors, osteoblasts (OPN+), vascular endothelium (EMCN+) cells were observed in all groups, indicating dynamic osteogenic activity. Macrophages, only M2, were observed in conditions producing fibrous tissue. Conclusions: In this newly developed non-wear-particle-related murine osseointegration failure model, the cellular composition of human and murine interfacial tissue implicates specific populations of fibroblasts in fibrous tissue formation and implies that these cells may derive from mesenchymal stem cells.

## 1. Introduction

The formation of an osseous tissue between the implant and the host bone interface (osseointegration) is crucial in the success of total joint arthroplasty [1]. The failure of such process results in formation of fibrous tissue at the bone–implant interface, resulting in the low strength and loosening of the implant [2], and it is among the most common reasons for revision arthroplasty [3,4]. This fibrous membrane is thought to be formed by synoviocytes that migrate from the joint space into the bone–implant interface. The migration of synoviocytes also leads to subsequent release of osteoclast-stimulating cytokines, such as prostaglandin E2, collagenase [5,6,7] and the receptor activator of nuclear factor kappaB ligand (RANKL) [8], which further contribute to bone resorption [7]. In addition to pro-resorptive cytokines secreted by the synoviocytes, hydrostatic pressure and fluid flow transmitted through the fibrous membrane in absence of wear particles also induce periprosthetic bone destruction [9,10,11,12]. Regardless of the mechanism, the extent of fibrous tissue formation has been shown to correlate with the likelihood of loosening of both cemented and uncemented total joint arthroplasty [13].

Histological analysis of the interfacial fibrous membrane from retrieved orthopedic implants showed differences in cell composition and cell distribution based on the etiology of such membrane [14]. Wear-particle-induced fibrous membrane is characterized by the presence of wear particles [15] and abundant macrophages with multinucleated foreign giant-cell infiltration occupying more than 20% of the surface area [16], with accumulation of wear particles in the cytoplasm of these macrophages and giant cells [14,16,17]. In contrast to the wear-particle-induced fibrous membranes, non-wear-particle-induced interface membranes are characterized by the absence of wear particles and are mainly composed of fibrous or connective tissue with a high collagen content and only few cell nuclei [14,16,17] and low numbers of polymorphonuclear cells (PMNs), macrophages, plasma cells and lymphocytes, occupying a total of less than 20% of the membrane surface area [16].

The lack of animal models recapitulating the key features of non-wear-particle-induced interface membranes has largely precluded the identification of either the mechanisms involved or the preclinical development of molecular therapies. Most models of interfacial fibrous membranes have focused on wear-particle-induced membranes [18]. In these models, after the implantation of metal or polymethylmethacrylate (PMMA) implants, particles (metal, ceramic, polyethylene (PE) or PMMA) were injected into joint cavities to mimic intra-articular particles caused by wear. These particles triggered abundant recruitment of inflammatory cells, including macrophages, giant cells and fibrous tissue, similar to the wear-particle-induced fibrous membrane seen in clinical samples [19,20].

To address the lack of animal models for non-wear-particle-induced membranes, in this paper, we reported murine models that could be used as surrogates for poorly osseointegrated implants (referred to in the remainder of the paper as the “Fibrous group”). We showed that reliable fibrous membranes could be formed by animals receiving a press-fitted PMMA implant (referred to in the remainder of the paper as the “PMMA subgroup”), a loosely fitted untextured titanium implant (referred to in the remainder of the paper as the “Smooth titanium subgroup”) and a loosely fitted rough titanium implant (referred to in the remainder of the paper as the “Overdrilled titanium subgroup”), all of which were implanted in the proximal tibia of mice. We then further showed the significant cellular composition difference between the aforementioned murine model and the interface area that was formed at the bone–implant interface of a previously established well-osseointegrated murine tibial titanium implant (referred to in the remainder of the paper as the “Control group”) [21]. We then showed the similarity between the murine membranes and the interface membranes around loosening implants of patients receiving revision surgery. We validated that this model could be used as a platform to study the mechanism of membrane formation and to find new methods to prevent implant loosening.

## 2. Materials and Methods

### 2.1. Study Design

With approval from our Institutional Animal Care and Use Committee (IACUC Protocol 2018-0001), 16-week-old female C57BL/6 mice (*n* = 32, Jackson Laboratory, Bar Harbor, ME, USA) received different implants/conditions on the right tibia to induce either implant osseointegration or osseointegration failure. All mice started to ambulate and bear weight with the operated knee immediately after recovery from anesthesia. All mice were euthanized at 2 weeks after implantation. The right tibiae were collected for analyses.

To validate the clinical relevance of our mouse model, under a protocol approved by our Institutional Review Board (no. 2019-0571), we used human interfacial tissues obtained from two patients during revision TKA for aseptic loosening. The diagnosis of aseptic loosening was confirmed by the presence of radiographic peri-implant lucency and confirmed intraoperatively with mechanically loose implants and the presence of peri-implant fibrotic tissue. A septic cause of loosening was ruled out using the criteria defined by the Musculoskeletal Infection Society [22]. The tissue of the first patient, who was a 45-year-old female, underwent revision surgery for aseptic loosening 2 years after primary total knee replacement. The tissue of the second patient, who was a 40-year-old male, underwent revision surgery for aseptic loosening 3 years after primary total knee arthroplasty. Tissues were collected from the interface between the polymethylmethacrylate (PMMA) cement and the bone in the medullary canal of proximal tibia.

### 2.2. Osseointegration Model

**Figure 1:**
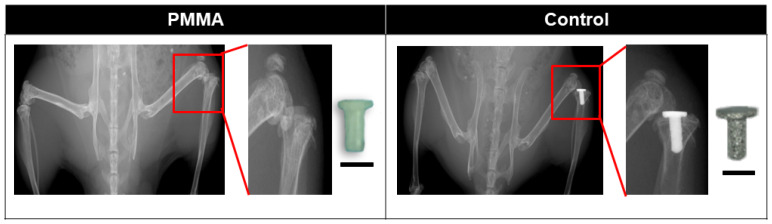
High resolution (Faxitron) images showing smooth polymethylmethacrylate (PMMA) or roughened titanium implant (Control) in right tibia. Scale bar = 1000 µm.

We used a model, which had been reported as representing successful osseointegration [21], as the Control (*n* = 11) group. Briefly, a 3D-printed titanium implant with smooth oval plateau (2.0 × 1.5 mm) and a 2.0 mm long intramedullary stem roughened with 40 µm diameter titanium spheres (manufactured by Smith&Nephew, London, UK) was press-fitted into a 0.9 mm hole drilled in the proximal tibia (Figure 1).

### 2.3. Osseointegration Failure Model

The following implants were used to induce osseointegration failure (osseointegration failure group):

(1) A PMMA (*n* = 11) implant (manufactured in our lab, Figure 1) with a smooth surface but otherwise the same dimensions and shape as the implant used in the Control group, modeling suboptimal implant material and poor surface topology.

(2) A titanium implant with both a smooth tibial plateau and intramedullary stem (Smooth-Ti, *n* = 5), modeling poor surface topology (manufactured by Smith&Nephew, London, UK).

(3) The same titanium implant as that in the Control group inserted into an overdrilled 1.4 mm hole in the proximal tibia (Overdrill, *n* = 5), modeling implant instability.

### 2.4. Biomechanical Testing

The strength of the bone–implant interface was determined by pullout testing, as described previously [21].

### 2.5. Histology

We used histology to identify the interfacial fibrous tissue (*n* = 2). Both mouse and human specimens were fixed in 10% formalin overnight and decalcified with 5M EDTA solution at 4 °C for 4 days. The implant was gently removed from the tibia before paraffin embedding [23]. Sections with 7 μm thickness were stained for hematoxylin and eosin (H&E) to show the structure of the interfacial tissue.

### 2.6. Immunofluorescence

We used immunofluorescence to determine the molecular composition of interfacial tissue (*n* = 3). The samples were fixed in 4% paraformaldehyde overnight at 4 °C and then decalcified with daily changes of 0.5M EDTA at 4 °C for 4 days [24]. All samples were embedded in an Optimal Cutting Temperature (OCT, Sakura, 4583, Torrance, CA, USA) compound and cut into 20 µm thick sections with a cryostat (Leica, Amsterdam, The Netherlands).

Immunofluorescence staining and analysis were performed as described previously [25]. The primary antibodies were mouse anti-CD68 (AB125212), anti-F4/80 (AB100790), anti-CD206 (AB64693), anti-PDGFRα (AB69569), anti-TCF4 (AB125212), anti-Leptin receptor (AB104403) and Anti-CD146 (AB75769) from Abcam (Cambridge, MA, USA); anti-CD206 (PA5-46994) and anti-iNOS (PA-030A) from ThermoFisher (Waltham, MA, USA); anti-F4/80 (NB600-404SS, Novus Biological, Centennial, CO, USA); anti-Arginase-1 (711765, Invitrogen, NY, USA); anti-Pu.1 (22585, Cell Signaling, Danvers, MA, USA); anti-Leptin receptor (AF497, R&D Systems, Minneapolis, MN, USA); Anti-Endomucin (sc65495, Santa Cruz Biotech, Santa Cruz, CA, USA); and anti-CD31 (LS-C34876, LSBio, Seattle, WA, USA). Primary antibodies were visualized with species-appropriate Alexa Fluor–coupled secondary antibodies (1:400 for 3 h, ThermoFisher). Nuclei were counterstained with 4′6-diamidino-2-phenylindole (DAPI).

All images were captured with a confocal microscope (LSM 880, Zeiss, Oberkochen, Germany) and reconstructed with ImageJ distribution Fiji (http://rsbweb.nih.gov/ij/, accessed on 9 September 2022) [26,27]. All immunofluorescence analyses were confirmed via at least one independent repeat.

### 2.7. Statistical Analysis

Maximum load was reported as mean ± 95% confidence. Statistical analysis was performed using a two-tailed, unpaired Student’s *t*-test with α = 0.05.

## 3. Results

### 3.1. Interface Strength Was Lower in the Fibrous Group than in the Control Group

The interface strength between bone and implant has previously been reported to be positively correlated with the degree of osseointegration [28]. We performed an implant pullout test [21], as previously reported, as an indicator of the degree of osseointegration. The mean maximum pullout load at week two after implantation in the PMMA subgroup was 2.8 ± 1.8 N, which was 87% lower than the value in the Control group (21 ± 4.6 N, *p* < 0.001). The implants in the Smooth titanium and Overdrilled titanium subgroups were grossly loose and therefore not subjected to the pullout test.

### 3.2. Presence of Fibrous Membrane Was Seen in the Fibrous Group Mice and Human Interface Membrane but Not in the Control Mice

**Figure 2:**
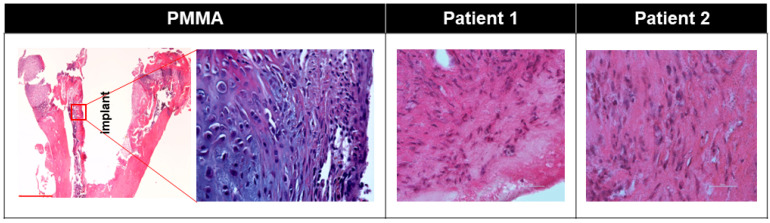
Histologic images (H&E) showing nuclei interspersed in fibrous matrix the bone-implant interfacial tissue of mouse received PMMA implant (scale bar = 500 μm) and human (scale bar = 50 μm) received cementless implant (patient 1) or cemented implant (patient 2). No wear particles or multinucleated giant cells observed.

To further explore the etiology of the difference in interface strength between the Control and Fibrous groups, we used histology to examine the interface area. In the Control group, only mineralized bone and no fibrous tissue was seen at the bone–implant interface (Figure 2). On the other hand, interface tissues consisting of a fibrous-rich connective tissue matrix were seen in the Fibrous group (Figure 2, Supplementary Figure S1).

Similarly, the interface membrane of both patients showed sparse cells interspersed in a collagen fiber-rich connective tissue matrix (Figure 2). No multinucleated foreign-body giant cells were seen in either of the human or murine interface membranes (Figure 2).

### 3.3. Abundant Fibroblasts Were Seen in the Fibrous Group and Human Interface Membranes but Not in the Control Mice

To determine the cellular composition of the Control and Fibrous groups, we stained for a series of markers for cell types potentially implicated in fibrotic responses [29]: fibroblast-associated cell markers (PDGFRα, TCF4, Pu1), osteoblast-associated cell markers (osteopontin (OPN), osterix (OSX)), osteoclast-associated cell markers (tartrate-resistant acid phosphatase (TRAP)), macrophage-associated cell markers (F4/80, CD68, Arginase 1, CD206, iNOS), MSC-associated cell markers (CD146, Leptin receptor) and endothelial-associated cell markers (endomucin (EMCN), CD31)).

To determine the presence of fibroblasts in the human and murine interface membranes, we performed immunofluorescence staining of PDGFRα+ TCF4+ [30,31] and PDGFRα+ Pu.1+ [32] as markers of fibroblasts and myofibroblasts, respectively.

**Figure 3:**
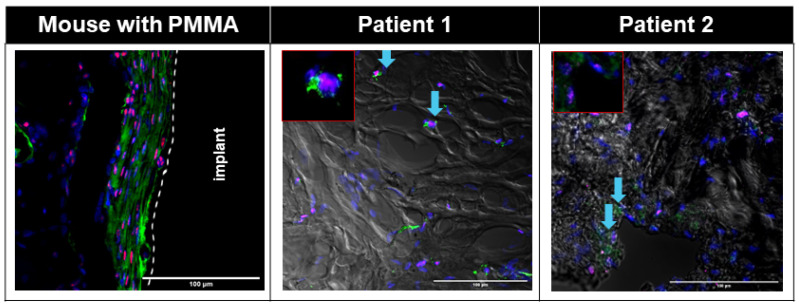
Immunofluorescent images showing abundant TCF4+ (red) PDGFRɑ+ (green) cells in both human and mouse interfacial fibrous tissue. Nuclei were counterstained with DAPI (blue). White dotted line: border of host tissue and implant. Patient fluorescent images were overlaid with corresponding transmitted bright field images. Blue arrow: TCF4+PDGFRɑ+ cells in patients.

**Figure 4:**
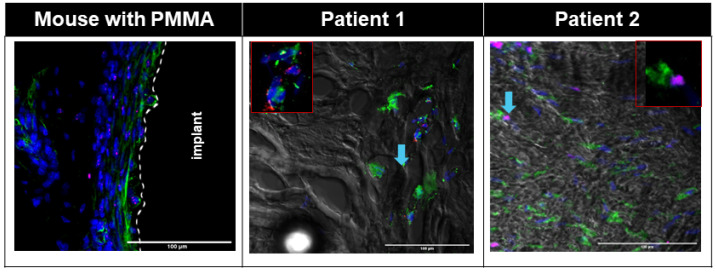
Immunofluorescent images showing pro-fibrotic Pu1+ (red) PDGFRɑ+ (green) cells in both human and mouse interfacial fibrous tissue. Nuclei were counterstained with DAPI (blue). White dotted line: border of host tissue and implant. Patient fluorescent images were overlaid with corresponding transmitted bright field images. Blue arrow: Pu1+PDGFRɑ+ cells in patients.

Small numbers of PDGFRα+ Pu1+ and PDGFRα+ TCF4+ cells were seen in the interface area of the Control group (Supplementary Figure S2), whereas large numbers of PDGFRα+ TCF4+ cells were seen in the interface membrane of the Fibrous group (Figure 3, Supplementary Figure S2), with all TCF4+ cells on the interface membrane also being PDGFRα+ (Figure 3, Supplementary Figure S2). Large numbers of cells in the murine interface membrane stained positive for TCF4. PDGFRα+ Pu1+ cells were also seen on the murine interface membrane (Figure 4, Supplementary Figure S3), albeit at a smaller density as compared to the PDGFRα+ TCF4+ cells (Figure 4).

Similar to the observation in the interface membrane of the Fibrous group, the interface membranes from both patients demonstrated an abundance of PDGFRα+ TCF4+ cells (Figure 3), with all TCF4+ cells also being PDGFRα+ (Figure 3). Further similarity between the murine and human interface membranes was demonstrated by the presence of PDGFRα+ Pu1+ fibroblasts in the interface membranes of both patients (Figure 4).

### 3.4. Osteopontin+ Cells Are Absent in the Fibrous Membrane Group

The differentiation of osteoblastic and pre-osteoblastic cells into mature osteoblasts and osteocytes has previously been studied using RT-PCR both in vivo and in vitro [33,34], which showed osterix (OSX), alkaline phosphatase (ALP), osteopontin (OPN), collagen 1 and osteocalcin (OCN) [35] as markers of osteoblastic lineage. In particular, osteopontin (OPN) [36] and osterix (OSX) are often considered as surrogate markers for pro-osteogenic cells [37] and pre-osteoblasts [38], respectively.

**Figure 5:**
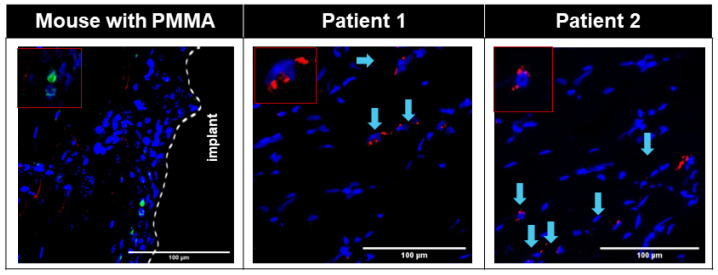
Immunofluorescent images showing OSX+ (red) cells in both human and mouse interfacial fibrous tissue; and extracellular OPN (green) only in mouse interfacial fibrous tissue. Nuclei were counterstained with DAPI (blue). White dotted line: border of host tissue and implant. Blue arrow: OSX+ cells in patients.

In the Control group, both OPN+ and OSX+ cells were observed at the bone–implant interface (Supplementary Figure S5). Extracellular OPN was also observed at the interface, which has previously been reported to represent recently mineralized tissue [39]. On the other hand, in the Fibrous group, we observed OSX+ cells deep inside the interface membrane, but not OPN+ cells (Figure 5, Supplementary Figure S4). Most of the OPN fluorescence in the murine samples was observed extracellularly and relatively distant from the actual bone–implant interface. In the human interface membranes, OPN+ cells were observed within the membrane, but not OSX+ cells (Figure 5).

### 3.5. Mononucleated but Not Multinucleated TRAP+ Cells Were Found Within All Interface Membranes

**Figure 6:**
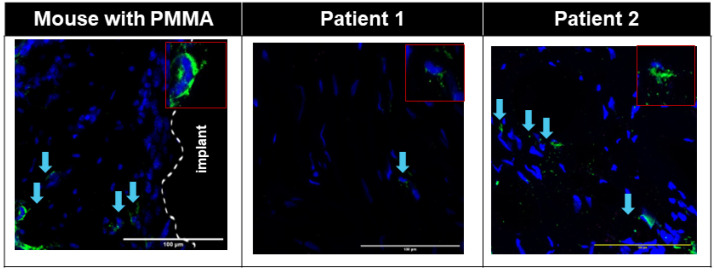
Immunofluorescent images showing sparse TRAP+ (green) cells, all mononucleated, in both human and mouse interfacial fibrous tissue. Nuclei were counterstained with DAPI (blue). White dotted line: border of host tissue and implant. Blue arrow: TRAP+ cells in patients.

Mononucleated TRAP+ cells have previously been shown to be early osteoclasts, which are already capable of performing bone resorption [38]. These mononucleated cells will subsequently fuse to form mature osteoclasts [38,39,40]. We observed the presence of several TRAP+ cells, all of which were mononucleated, in the Control group, Fibrous group and the human samples (Figure 6, Supplementary Figure S5). In the human interface membranes, all TRAP+ cells were embedded within the fibrous membrane (Figure 6). On the other hand, in the Fibrous group, while some TRAP+ cells were embedded within the fibrous membrane, some cells were located deep in the membrane, into the trabecular bone region (Figure 6, Supplementary Figure S5). The presence of early osteoclasts in both murine and human samples could indicate an active attempt of bone resorption/remodeling surrounding the fibrous membrane.

### 3.6. Both CD146+ and LepR+ Progenitor Cells Were Observed in Human and Murine Interface Membranes

During the formation of new mineralized tissues and fibrous tissues, progenitor cells are thought to be different effector cells that will form either mineralized tissues or fibrous tissues [41]. To find out the presence of progenitor cells (mesenchymal stem cells or skeletal stem cells) in the human and murine interface membranes, we performed immunofluorescent staining for CD146 [42,43], PDGFRα [43] and Leptin receptor (LepR, [44]), which were shown to be positively expressed by progenitor cells. LepR+ PDGFRα+ cells were reported in the MSC-enriched bone marrow stromal cells [43]. Due to their association with vasculature, LepR+ MSCs are considered to be a population of BM-resident MSCs [44].

**Figure 7:**
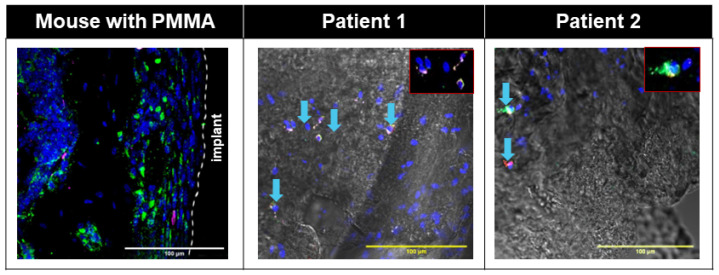
Immunofluorescent images showing abundant Leptin R^+^ (LepR^+^, green) and sparse CD146^+^ cells in mouse interfacial fibrous tissue; and sparse LepR^+^ (magenta) and PDGFRa^+^ (green) cells in human interfacial fibrous tissue. Nuclei were counterstained with DAPI (blue). White dotted line: border of host tissue and implant. Patient fluorescent images were overlaid with corresponding transmitted bright field images. Blue arrow: PDGFRa^+^LepR^+^ cells in patients.

**Figure 8:**
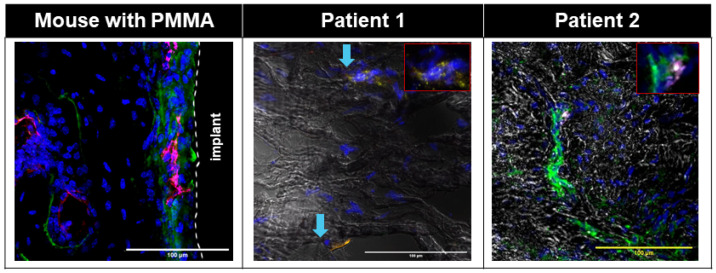
Immunofluorescent images showing CD146^+^ (magenta), PDGFRa^+^ (green), and Endomucin^+^ (red) cells in mouse interfacial fibrous tissue; and CD146^+^(red) and PDGFRa^+^(green) cells in human interfacial fibrous tissue. Nuclei were counterstained with DAPI (blue). White dotted line: border of host tissue and implant. Patient fluorescent images were overlaid with corresponding transmitted bright field images. Blue arrow: CD146^+^LepR^+^ cells in patients.

In both human and mouse membranes, we observed minimum overlap between LepR+ cells and CD146+ cells (Figure 7 and Figure 8, Supplementary Figures S6 and S7). Some CD146+ cells in the murine interface membrane overlapped with PDGFRα+ cells, which likely corresponds to the progenitor cells (Figure 7 and Figure 8, Supplementary Figures S6 and S7). On the other hand, in the Control group, there was a minimal number of LepR+ in the interface area but relative abundance of CD146+PDGFRα+ cells (Supplementary Figures S6 and S7).

### 3.7. Macrophage Compositions in Murine and Human Interface Membranes

Macrophages have been shown to play an important role during fracture repair, osteophyte formation [45], bone destruction [46] and osteoporosis [47] through the secretion of osteoactive cytokines [48], such as matrix metalloproteinase [46] and bone morphogenic protein [48]. Furthermore, the maintenance of osseous tissue macrophages was likely sustained by osteoblast production of CSF-1 [49]. To find out whether macrophages were present in the human and murine interface membranes, we performed immunofluorescence staining for CD68 for human [50] and F4/80 for murine [51] membranes as general markers of macrophages. For markers of M2 polarized macrophages, we used Arginase-1 for human [52] and CD206 for murine staining [52]. iNOS was used as a representative marker of M1 macrophages [53].

**Figure 9:**
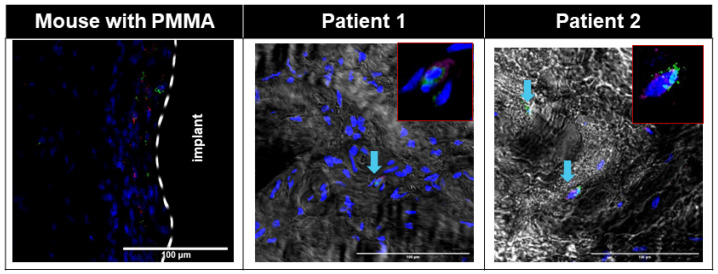
Immunofluorescent images showing sparse macrophages (red, F4/80 for mice and CD68+ for human), mostly M2 (green, CD206 for mice and Arginase1 for human), in both human and mouse interfacial fibrous tissue. Nuclei were counterstained with DAPI (blue). White dotted line: border of host tissue and implant. Patient fluorescent images were overlaid with corresponding transmitted bright field images. Blue arrow: M2 macrophages in patients.

The interface membrane in the Fibrous group showed a small number of F4/80+ CD206+ cells (Figure 9, Supplementary Figure S8), and all F4/80+ cells were also CD206+ (Figure 9, Supplementary Figure S8). The interface area of the Control group also showed very few F4/80+ cells, and most F4/80+ cells were outside the interface region (Supplementary Figure S8).

Similar to the Fibrous group, the interface membranes of both patients showed a small number of CD68+Arginase-1+ cells (Figure 9). In fact, all CD68+ cells were also Arginase-1+. No CD68+ iNOS+ cells were found on the murine and human interface membranes.

## 4. Discussion

In the present study, we presented murine models of non-wear-particle-induced, poorly osseointegrated implants using a press-fitted PMMA implant, a smooth titanium implant and an overdrilled rough titanium implant. In the current study, the PMMA and smooth titanium implants were used to mimic the clinical conditions of poor initial fixation, poor mechanical interlocking and micromotion between bone-cemented implant and bone-uncemented implant, respectively. The overdrilled rough titanium implant was used to mimic increased micromotion while still allowing potential mechanical interlocking between the bone and micropores on the implant. Interestingly, analysis of the interface membranes in all three models showed the formation of fibrous interface membranes with similar cellular compositions.

Unlike wear-particle-induced fibrous interface membrane, which is often characterized by a large amount of macrophage infiltration with multinucleated foreign-body giant cells, non-wear-particle-induced interface membrane is often characterized by a lack of wear particles, an abundance of connective tissue, relatively sparse nuclei and low amount of macrophage infiltration [16]. Histology of the membranes of both patients in our studies corroborated the previous findings (Figure 2). Minimum macrophage infiltration in the human interface membrane was demonstrated by the sparse number of CD68+ cells (Figure 9). All CD68+ cells in both patients were also Arginase-1+, which is commonly seen in M2 macrophages [54]. Similarly, in murine interface membranes, all F4/80+ cells were also CD206+ (Figure 9, Supplementary Figure S8), which is also commonly seen in the M2 macrophage subtype [55,56,57]. In contrast to M1 macrophages, which are generally considered pro-inflammatory [54], M2 macrophages negatively regulate pro-inflammatory cytokines and induce the production of anti-inflammatory cytokines, such as Il-4, IL10 and TGF-β [58,59]. Furthermore, M2 macrophages are crucial for repair mechanisms, which include ECM depositions, macrophage adhesion and the removal of cell debris [55,56,57].

In addition to a low level of macrophage infiltration in the membrane, the presence of fibroblasts is also characteristic of non-wear-particle-induced membranes [16]. PDGFRα [30], TCF4 [31] and Pu.1 [32] have previously been reported as positive markers of fibroblasts. In both murine and human interface membranes, we observed an abundance of TCF4+PDGFRα+ cells, which was previously shown to positively correlate with the degree of fibrosis in both acute and chronic injuries [60]. TCF4 is one of the major regulators of fibroblast-to-myofibroblast differentiation [31], which, upon injury, expresses a large amount of extracellular matrix, resulting in the formation of fibrotic tissues in several organs, such as the muscles and liver [61,62,63,64]. Furthermore, we also observed the presence of PDGFRα+ Pu1+ fibroblasts in the interface membranes of both patients (Figure 4) and mice (Figure 4, Supplementary Figure S3), which were previously shown to have the highest enrichment of pro-fibrotic fibroblasts [32]. The activation of Pu.1 in human fibroblasts is induced by a transition of resting fibroblasts from healthy donors to pro-fibrotic phenotypes with the upregulation of collagen release, α-SMA and actin [32]. In addition, the inhibition of Pu.1 both prevented fibrosis and induced regression of pre-established fibrosis [32].

In addition to macrophages and fibroblasts, previous studies of retrieved human clinical samples also demonstrated the presence of osteoblasts [65], TRAP+ cells [66] and endothelial cells [16,17] within the membranes. We observed the presence of OPN+ cells in both patients’ membranes (Figure 5), which has previously been seen in pro-osteogenic cells/osteoblasts [67]. The presence of osteoblasts was also seen in human interface membranes [65], which might indicate the host’s attempt at bone formation and repair in the presence of bone resorption. Interestingly, while we observed the presence of extracellular OPN cells beneath the murine interface membrane, which is usually seen in newly formed mineralized tissue [67], we did not observe any OPN+ cells within or around the interface membrane (Figure 5, Supplementary Figure S5). Instead, we observed OSX+ cells around the murine interface membrane (Figure 5, Supplementary Figure S4), which is an osteoblast-specific transcription factor expressed during differentiation of pre-osteoblasts into mature osteoblasts [68]. The presence of OSX+ cells beneath the interface membrane could indicate that the host is attempting to remodel the fibrous interface into mineralized tissue. Whether this attempt at remodeling fibrous tissue to mineralized tissue is successful or not is currently unknown and will be the subject of future studies.

In both of our murine and human interface membranes, we observed the presence of mononucleated TRAP+ cells (Figure 6, Supplementary Figure S5), which has previously been seen in mononucleated pre-osteoclasts [69] and mononucleated monocytes [70]. A possible reason for the absence of multinucleated TRAP+ cells in the interface membrane is that osteoclasts tend to be present right at the junction between the bone and the membrane, not within the membrane itself [71].

In addition to the stromal cells and immune cells, we also observed CD146+PDGFRα+ [72] and LepR+PDGFRα+ [73] cell populations in both human and murine interface membranes (Figure 7 and Figure 8, Supplementary Figures S6 and S7), which have previously been reported to be enriched in mesenchymal stem cell populations. MSCs are multipotent and can differentiate in vivo toward skeletal lineages, such as osteoblasts, adipocytes and chondrocytes, as well as toward fibroblastic stromal cells [42,74]. CD146+ bone marrow mesenchymal stem cells (BM-MSCs) are located in perivascular regions [72] and have been associated with both increasing [75] and decreasing fibrous tissue formation in vitro and in vivo [42,76]. LepR+ MSC cells have also been previously shown to play crucial roles in osseous tissue formation and initiation of fibrosis through further differentiation into myofibroblasts and activation of PDGFRα signaling [77,78]. The presence of both LepR+PDGFRα+ and CD146+PDGFRα+ cells in human and mouse fibrous membranes might indicate that, while under the correct circumstances, these MSCs form new osseous tissue, and these cells may become pro-fibrotic MSCs. However, the exact fate of these so-called MSCs and their role in the formation and remodeling of the fibrous membrane will need to be further elucidated.

We observed the presence of EMCN+ cells in our murine interface membrane (Figure 8), which were shown to be highly expressed in tissues that undergo angiogenesis and osteogenesis [79]. This finding, combined with the aforementioned finding of the presence of both LepR+PDGFRα+ and CD146+PDGFRα+ cells, raises an interesting possibility that the hosts are attempting to remodel the fibrous membrane into bone.

One limitation of our study is that we compared the acute interface membrane from our murine model with the chronic interface membrane from patients. Human samples of early interface membrane are very difficult to obtain, given that aseptic loosening is not symptomatic until months to years later [80]. Interestingly, despite the aforementioned limitation, the interface membranes from all three murine models and the two patients showed similar cellular compositions.

In conclusion, we demonstrated here a novel murine model of non-wear-particle-induced fibrous membrane. We found a large number of similarities in cell compositions between the human and murine interface membranes. Both human and murine interface membranes showed a small amount of macrophage infiltration, a large amount of pro-fibrotic fibroblasts and the presence of TRAP+, pro-osteogenic and vascular cells. In addition, we also observed LepR+PDGFRα+ and CD146+PDGFRα+ cell populations, which were previously reported to be MSCs. The presence of MSCs and osteoblastic lineage cells in both human and murine membranes points toward an active effort by the hosts toward remodeling of the fibrous interface membrane and/or osseous tissue formation at the interface.

## 5. Conclusions

Here, we showed a novel murine model of non-wear-particle-induced fibrous membrane. We found a large number of similarities in cell compositions between the human and murine interface membranes. Both human and murine interface membranes showed a small amount of macrophage infiltration, a large amount of pro-fibrotic fibroblasts and the presence of TRAP+, pro-osteogenic and vascular cells. In addition, we also observed LepR+PDGFRα+ and CD146+PDGFRα+ cell populations, which were previously reported to be MSCs. The presence of MSCs and osteoblastic lineage cells in both human and murine membranes points toward an active effort by the hosts toward remodeling of the fibrous interface membrane and/or osseous tissue formation at the interface.

## Data Availability

The data supporting the findings of this study are available from the corresponding author X.Y. upon reasonable request.

## Supplementary Materials

The following supporting information can be downloaded at https://www.mdpi.com/article/10.3390/biomimetics9110673/s1.
