# Supplementary figures and images for: A Murine Model of Non-Wear-Particle-Induced Aseptic Loosening
Source: Biomimetics (Basel). 2024 Nov 4;9(11):673. doi: 10.3390/biomimetics9110673 (PMC11592190; doi:10.3390/biomimetics9110673)

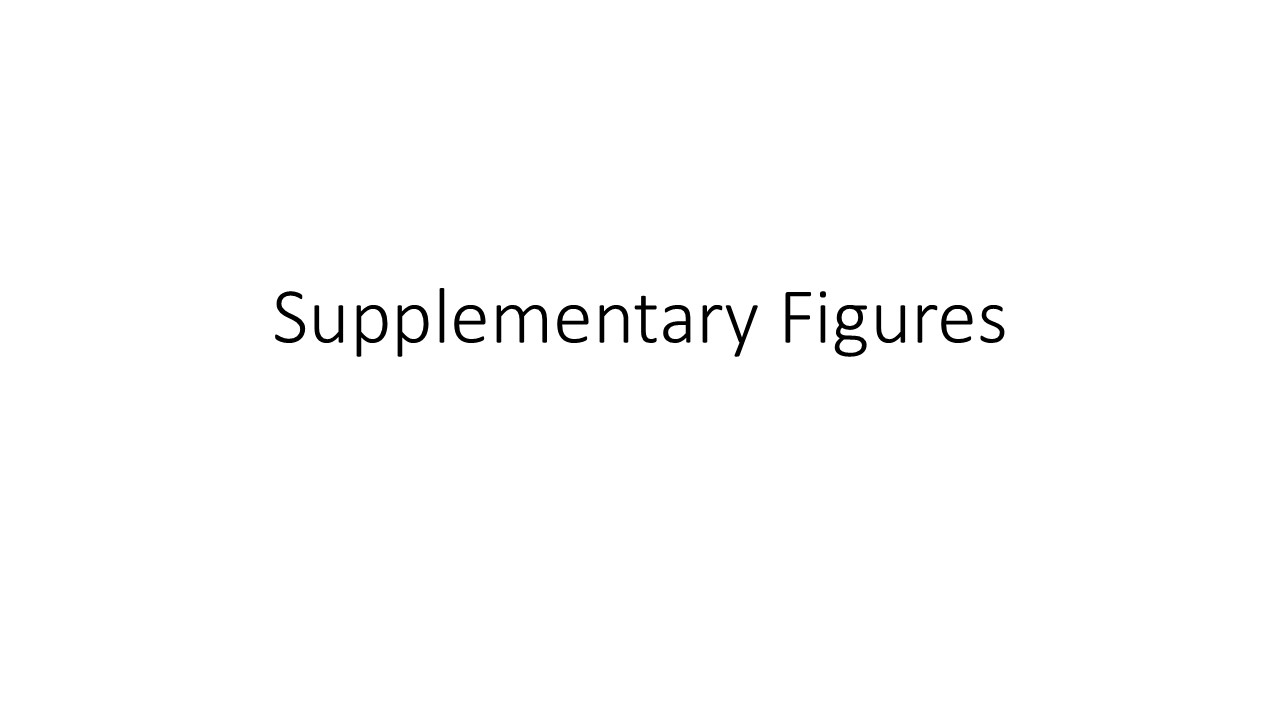

Supplement: Supplementary file 1 [file biomimetics-09-00673-s001.zip › Supplementary Figures_FinalManuscript/Slide1.JPG]

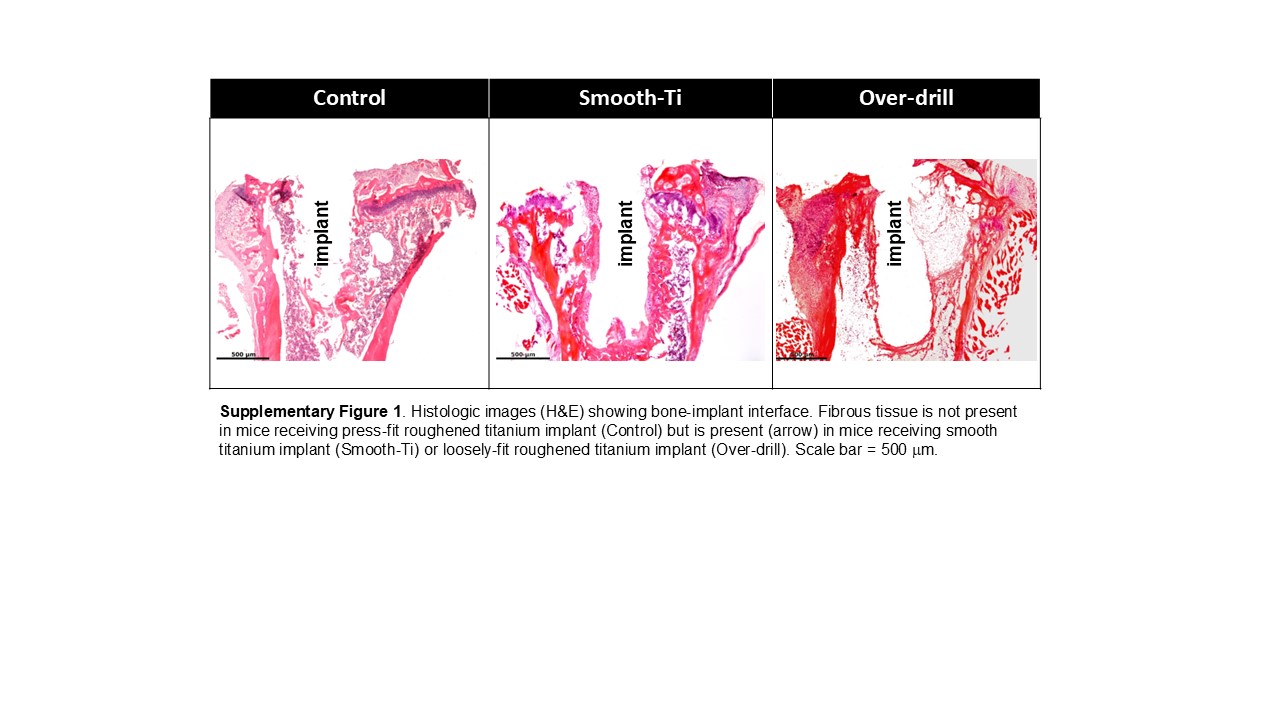

Supplement: Supplementary file 1 [file biomimetics-09-00673-s001.zip › Supplementary Figures_FinalManuscript/Slide2.JPG]

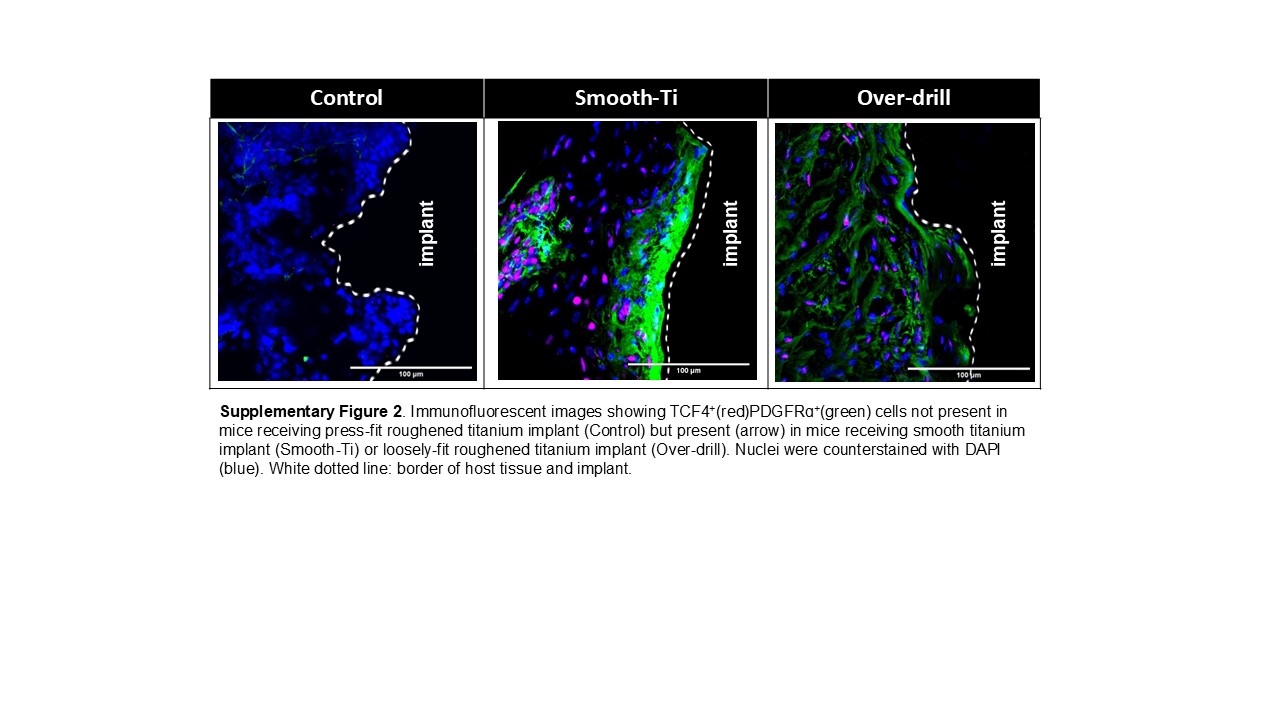

Supplement: Supplementary file 1 [file biomimetics-09-00673-s001.zip › Supplementary Figures_FinalManuscript/Slide3.JPG]

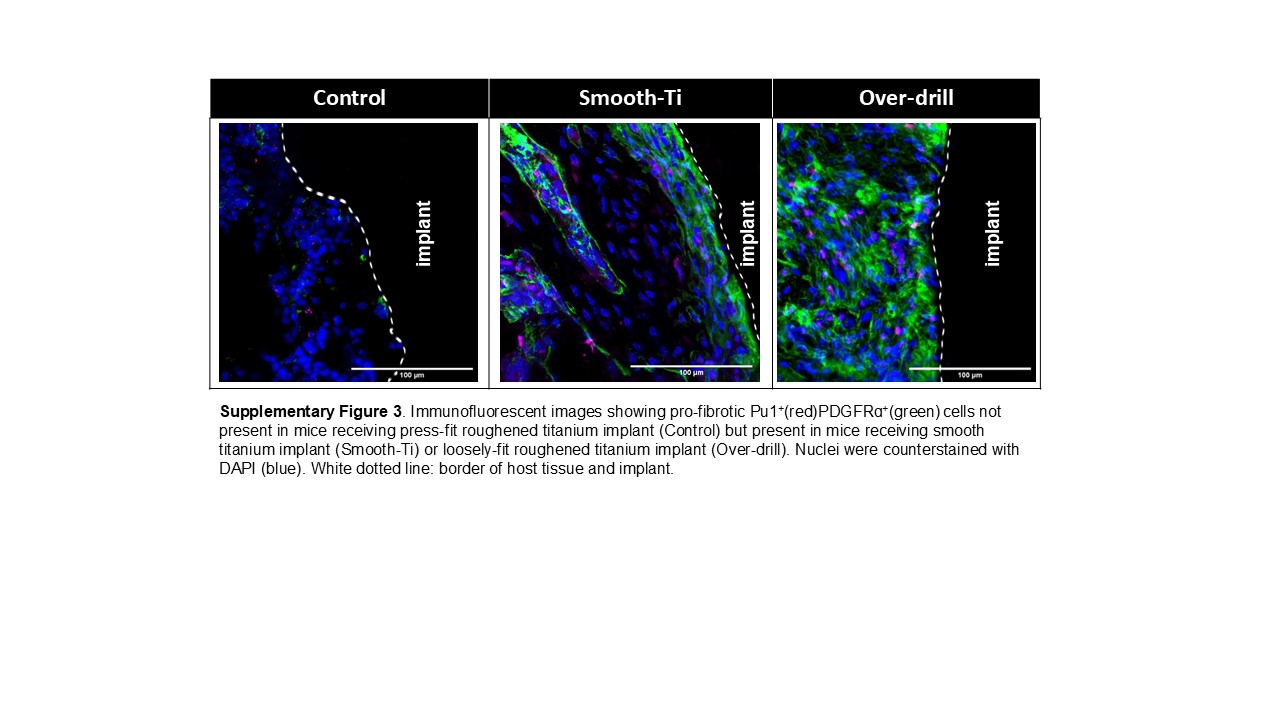

Supplement: Supplementary file 1 [file biomimetics-09-00673-s001.zip › Supplementary Figures_FinalManuscript/Slide4.JPG]

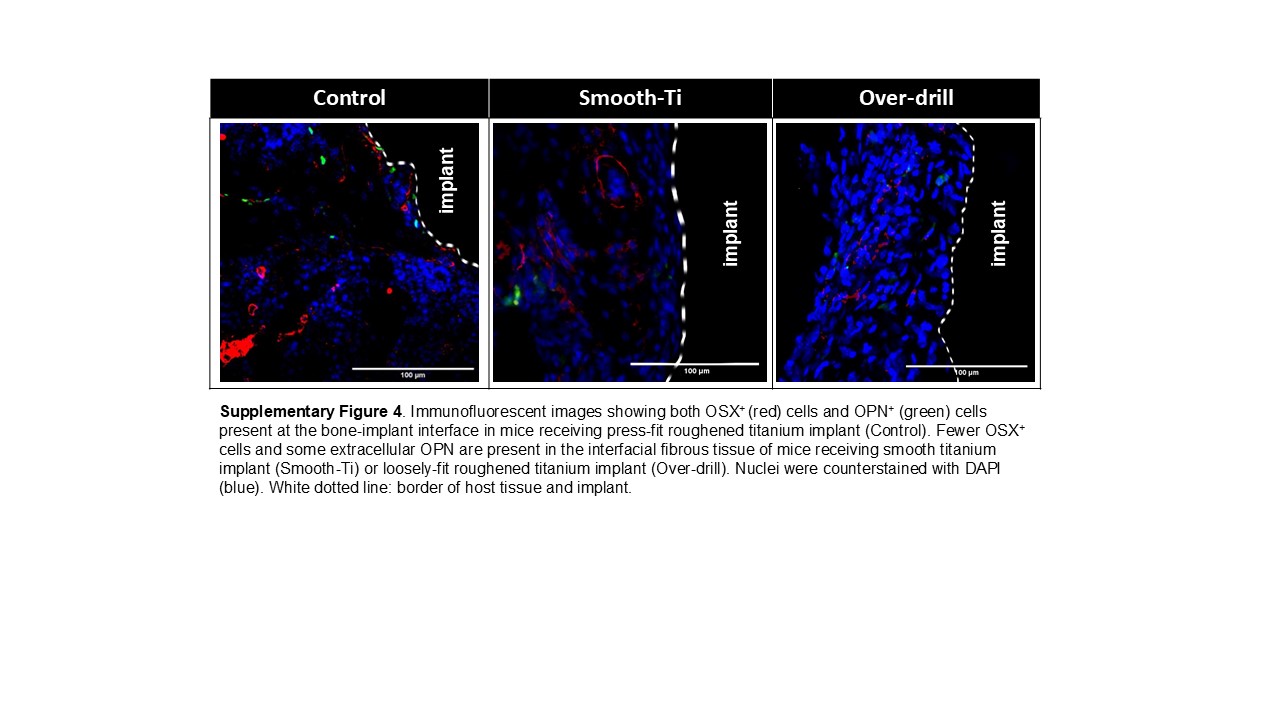

Supplement: Supplementary file 1 [file biomimetics-09-00673-s001.zip › Supplementary Figures_FinalManuscript/Slide5.JPG]

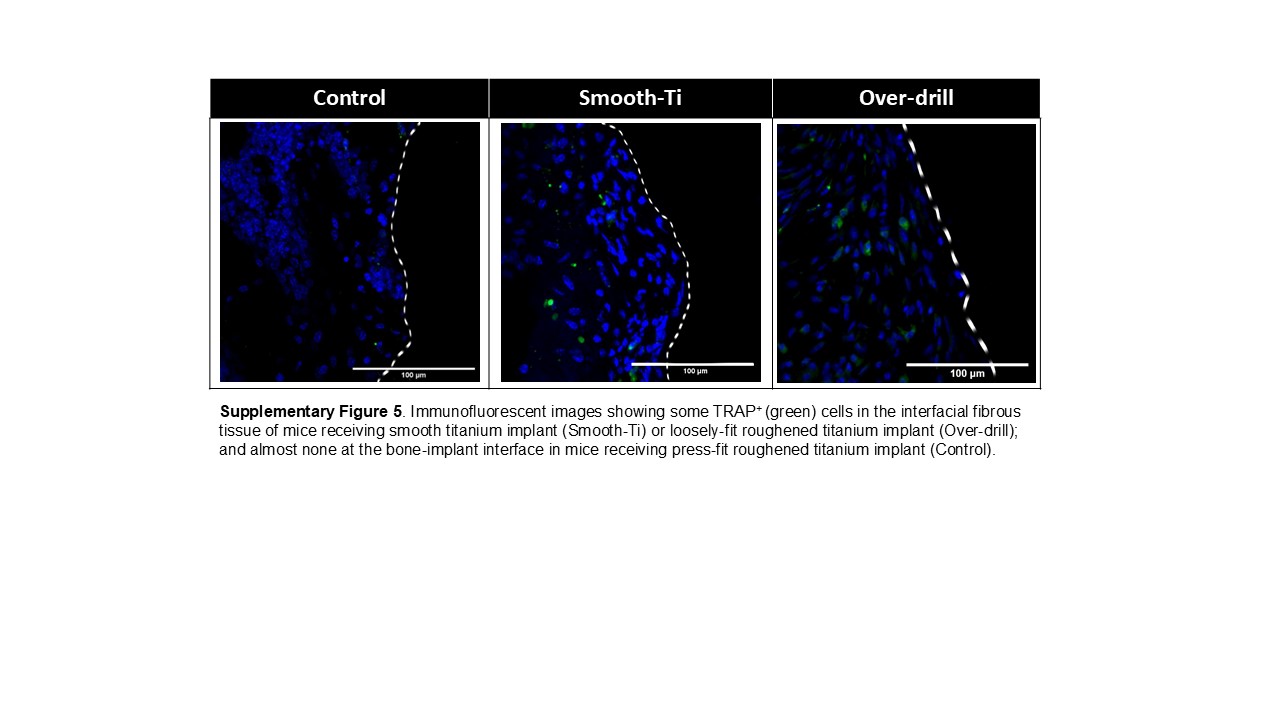

Supplement: Supplementary file 1 [file biomimetics-09-00673-s001.zip › Supplementary Figures_FinalManuscript/Slide6.JPG]

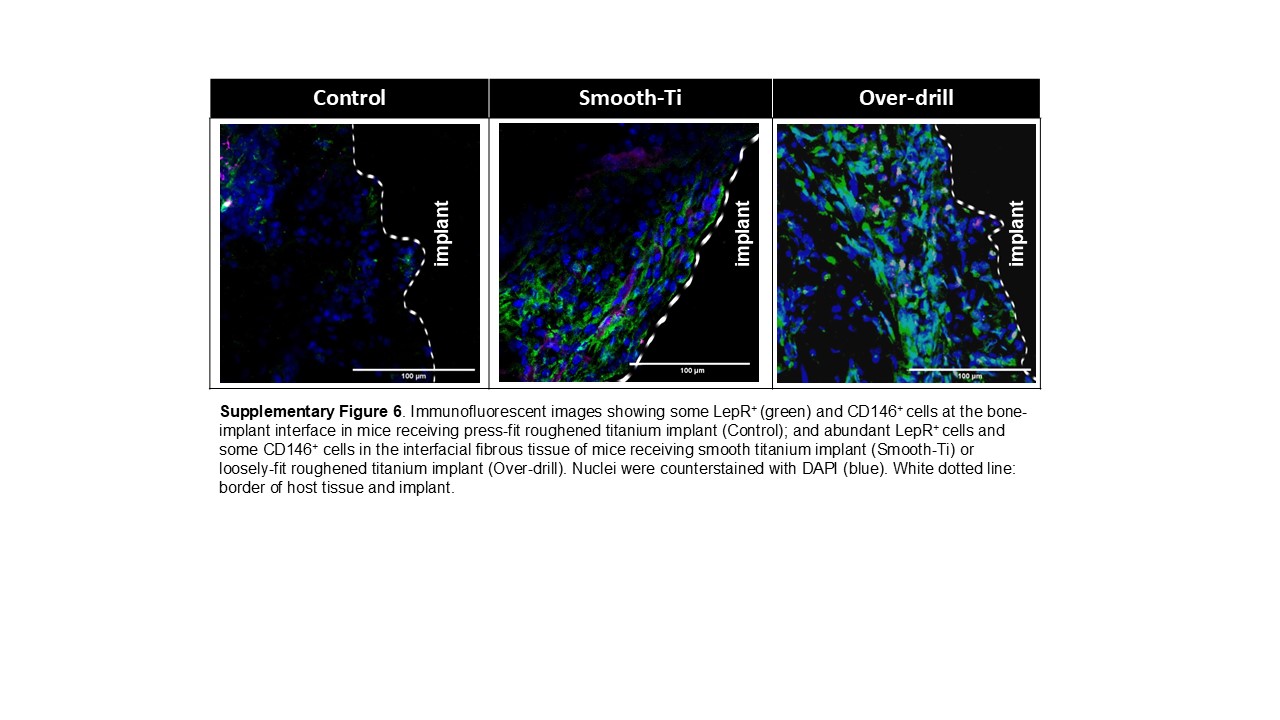

Supplement: Supplementary file 1 [file biomimetics-09-00673-s001.zip › Supplementary Figures_FinalManuscript/Slide7.JPG]

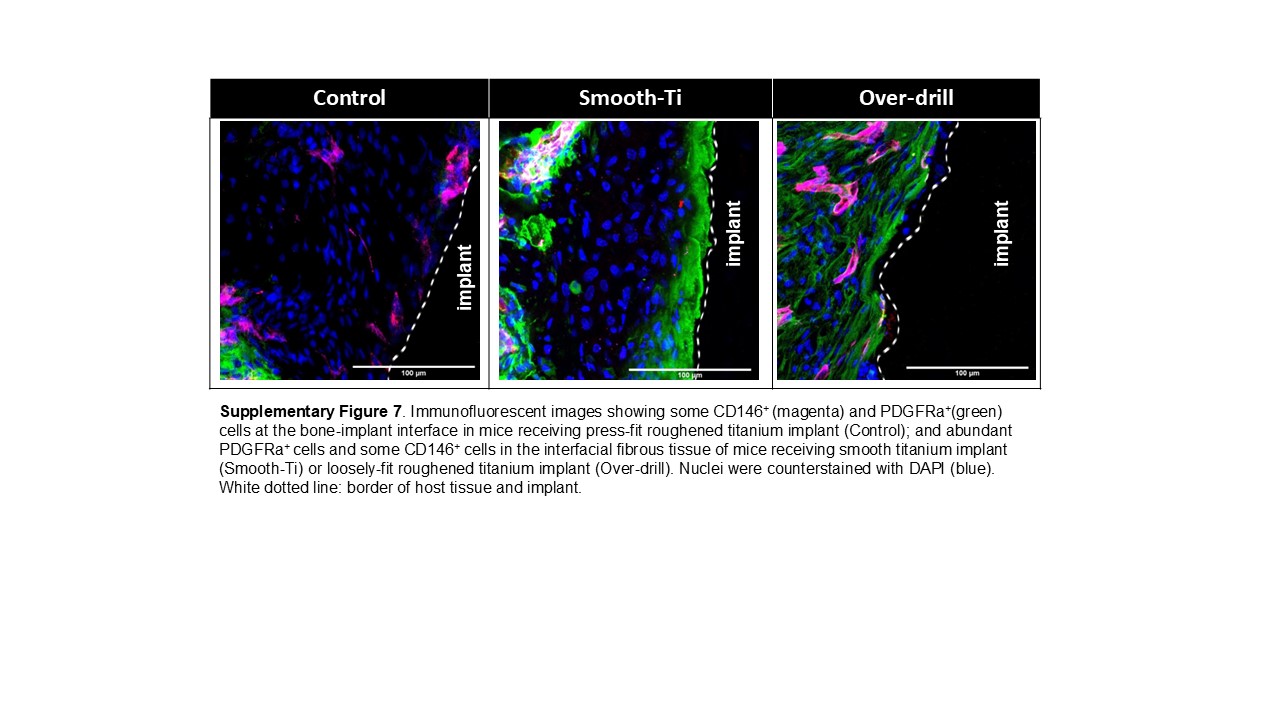

Supplement: Supplementary file 1 [file biomimetics-09-00673-s001.zip › Supplementary Figures_FinalManuscript/Slide8.JPG]

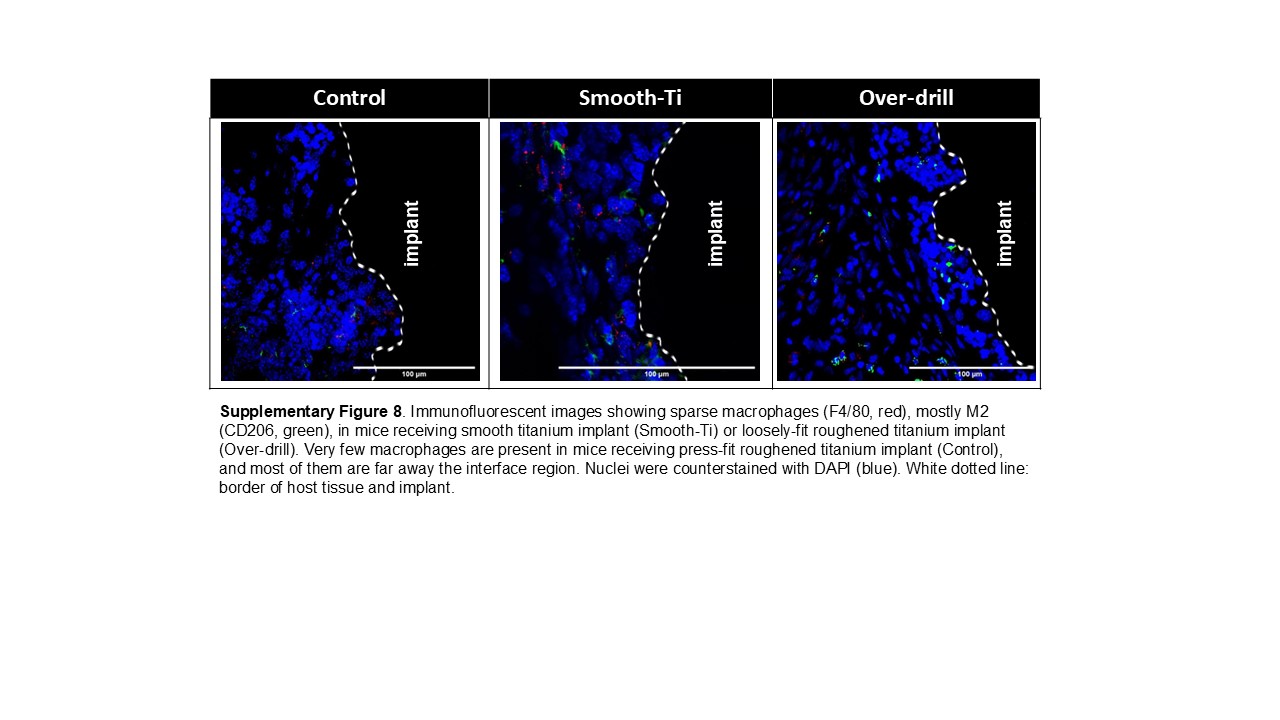

Supplement: Supplementary file 1 [file biomimetics-09-00673-s001.zip › Supplementary Figures_FinalManuscript/Slide9.JPG]
